# Supplementary material for: Contrast-enhanced ultrasound for the diagnosis of acute pyelonephritis in pediatric patients with urinary tract infection: A feasibility study
Source: PLoS One. 2023 Apr 5;18(4):e0284016. doi: 10.1371/journal.pone.0284016 (PMC10075446; doi:10.1371/journal.pone.0284016)
Supplement: Attachment — Submitted filename: Response to Reviewers.docx [file pone.0284016.s001.docx]

PONE-D-22-08175

Contrast-enhanced ultrasound for the diagnosis of acute pyelonephritis in pediatric patients with urinary tract infection

PLOS ONE

Dear Dr. Lee,

Thank you for submitting your manuscript to PLOS ONE. After careful consideration, we feel that it has merit but does not fully meet PLOS ONE’s publication criteria as it currently stands. Therefore, we invite you to submit a revised version of the manuscript that addresses the points raised during the review process.

Please submit your revised manuscript by Jul 18 2022 11:59PM. If you will need more time than this to complete your revisions, please reply to this message or contact the journal office at [plosone@plos.org](mailto:plosone@plos.org). When you're ready to submit your revision, log on to <https://www.editorialmanager.com/pone/> and select the 'Submissions Needing Revision' folder to locate your manuscript file.

Please include the following items when submitting your revised manuscript:

- A rebuttal letter that responds to each point raised by the academic editor and reviewer(s). You should upload this letter as a separate file labeled 'Response to Reviewers'.
- A marked-up copy of your manuscript that highlights changes made to the original version. You should upload this as a separate file labeled 'Revised Manuscript with Track Changes'.
- An unmarked version of your revised paper without tracked changes. You should upload this as a separate file labeled 'Manuscript'.

If you would like to make changes to your financial disclosure, please include your updated statement in your cover letter. Guidelines for resubmitting your figure files are available below the reviewer comments at the end of this letter.

If applicable, we recommend that you deposit your laboratory protocols in [protocols.io](http://protocols.io/) to enhance the reproducibility of your results. Protocols.io assigns your protocol its own identifier (DOI) so that it can be cited independently in the future. For instructions see: <https://journals.plos.org/plosone/s/submission-guidelines#loc-laboratory-protocols>. Additionally, PLOS ONE offers an option for publishing peer-reviewed Lab Protocol articles, which describe protocols hosted on [protocols.io](http://protocols.io/). Read more information on sharing protocols at <https://plos.org/protocols?utm_medium=editorial-email&utm_source=authorletters&utm_campaign=protocols>.

We look forward to receiving your revised manuscript.

Kind regards,

Israel Franco, M.D.
Academic Editor
PLOS ONE

Journal Requirements:

When submitting your revision, we need you to address these additional requirements.

1. Please ensure that your manuscript meets PLOS ONE's style requirements, including those for file naming. The PLOS ONE style templates can be found at

<https://journals.plos.org/plosone/s/file?id=wjVg/PLOSOne_formatting_sample_main_body.pdf> and

<https://journals.plos.org/plosone/s/file?id=ba62/PLOSOne_formatting_sample_title_authors_affiliations.pdf>

2. In your statement, please include the full name of the IRB or ethics committee who approved or waived your study.

Additional Editor Comments :

Please see the comments from the reviewers and make appropriate revisions to the paper based on their comments.

if there are additional subjects that can be added to the paper please consider adding the information.


[Note: HTML markup is below. Please do not edit.]

Reviewers' comments:

Reviewer's Responses to Questions

**Comments to the Author**

**1. Is the manuscript technically sound, and do the data support the conclusions?**

**The manuscript must describe a technically sound piece of scientific research with data that supports the conclusions. Experiments must have been conducted rigorously, with appropriate controls, replication, and sample sizes. The conclusions must be drawn appropriately based on the data presented.**

Reviewer #1: Yes

Reviewer #2: Partly

**2. Has the statistical analysis been performed appropriately and rigorously?**

Reviewer #1: I Don't Know

Reviewer #2: Yes

**3. Have the authors made all data underlying the findings in their manuscript fully available?**

The [PLOS Data policy](http://www.plosone.org/static/policies.action#sharing) requires authors to make all data underlying the findings described in their manuscript fully available without restriction, with rare exception (please refer to the Data Availability Statement in the manuscript PDF file). The data should be provided as part of the manuscript or its supporting information, or deposited to a public repository. For example, in addition to summary statistics, the data points behind means, medians and variance measures should be available. If there are restrictions on publicly sharing data—e.g. participant privacy or use of data from a third party—those must be specified.

Reviewer #1: No

Reviewer #2: Yes

**4. Is the manuscript presented in an intelligible fashion and written in standard English?**

PLOS ONE does not copyedit accepted manuscripts, so the language in submitted articles must be clear, correct, and unambiguous. Any typographical or grammatical errors should be corrected at revision, so please note any specific errors here.

Reviewer #1: Yes

Reviewer #2: Yes

**5. Review Comments to the Author**

Please use the space provided to explain your answers to the questions above. You may also include additional comments for the author, including concerns about dual publication, research ethics, or publication ethics. (Please upload your review as an attachment if it exceeds 20,000 characters)

**Reviewer #1: The authors attempt to show that CEUS can be used to diagnose acute pyelonephritis. We all would like to have minimal invasive imaging for our families. The mention most issues of their findings in their limitations. They do have a very small enrollment for a prospective study. While they tell us their inclusion and exclusion criteria they do not tell us the number of patient seen for a febrile in the same time period.**

🡪 Thank you for your thoughtful comments. We fully agree with your comments.

A total of 72 patients with suspected febrile urinary tract infection underwent a renal ultrasound during the study period, of which 21 patients participated in this study. Because this study was approved only as a clinical trial for ultrasound contrast agent, it was difficult to obtain written consent from the subjects' parents, and as a result, the number of participants was small. However, the patients who participated in this study did not show any side effects, such as headache, nausea, or skin erythema, even though they were infants at 9.0 months of age (2.0 months – 61.0 months). Therefore, these data could be regarded as an additional value to the safety of ultrasound contrast agents. As you mentioned, the study is insufficient to recommend CEUS alone for the diagnosis of APN due to the small number of subjects; however, we think it will be useful for researchers interested in the role and safety of ultrasound contrast agents in the diagnosis of APN in pediatric UTI patients.

**As they mention it is also problematic that not all patients got a DMSA scan but rather a selected view. As a follow up study it would sure be interesting about the long term findings by possibly repeating the tests after >12 month. It would also be interesting if the patients had VUR. In summary I do think the data is reportable with the limitations that they mention. The data is not enough to recommend CEUS alone but it is a start to possibly replace DMSA scans in the future.**

🡪 Thank you for your thoughtful comments. We fully agree with your comments.

A total of six patients had a follow-up ultrasound instead of a DMSA scan after CEUS (490.0 ± 168.5 days), of which only one showed abnormal findings in the grayscale ultrasound, such as parenchymal atrophy. In this patient, the initial grayscale ultrasound showed diffusely increased parenchymal echotexture and slight pelvic dilatation of both kidneys, but no focal lesions were found on CEUS. A follow-up voiding cystourethrography in this patient revealed vesicoureteral reflux, and the parenchymal damage was confirmed in the follow-up ultrasound. Therefore, this case could be regarded as a false-negative case of CEUS.

As noted in the manuscript, CEUS may give false-negative results if there are diffuse renal parenchymal changes, as in this patient, and it might be due to the operators' insufficient experience detecting the perfusion abnormality. The examinator will be able to see diffuse pathological changes only when they are familiar with the condition of normal renal perfusion in CEUS. It is necessary to accumulate more experience with diffuse renal perfusion abnormalities in the future.

The remaining five patients showed normal ultrasound findings, which indirectly confirmed that there was no APN at the time of CEUS. A follow-up ultrasound was also performed on three patients who underwent a DMSA scan, and a follow-up ultrasound confirmed the atrophy or cortical thinning remained in the area where cortical defects were observed in the DMSA scan.

As you mentioned, it is problematic that not all patients got a DMSA scan, which leads to the selected view. A follow-up ultrasound after CEUS (490.0 ± 168.5 days) was further investigated to overcome an insufficient number of DMSA scan results and to compensate for this study's limitation. We tried to improve the reliability of the negative results of CEUS by adding cases with normal findings on follow-up ultrasound. Additionally, a CEUS false-negative case was found in a patient with VUR, further adding an explanation of the false-negative risk during the CEUS examination.

**🡪 We revised and added the manuscript as follows:**

Materials and Methods

Participants: A total of 72 patients with suspected febrile urinary tract infection underwent a renal ultrasound during the study period, of which 21 patients participated in this study.

Data Analysis: The same analysis of follow-up grayscale images was performed if the follow-up ultrasound was performed instead of a DMSA scan.

Results

A total of six patients had a follow-up ultrasound instead of a DMSA scan after CEUS (490.0 ± 168.5 days), of which only one showed abnormal findings in the grayscale ultrasound, such as parenchymal atrophy. In this patient, the initial grayscale ultrasound showed diffusely increased parenchymal echotexture and slight pelvic dilatation of both kidneys, but no focal lesions were found on CEUS. A follow-up voiding cystourethrography in this patient revealed vesicoureteral reflux, and the parenchymal damage was confirmed in the follow-up ultrasound. Therefore, this case could be regarded as a false-negative case of CEUS. The remaining five patients showed normal ultrasound findings, which indirectly confirmed that there was no APN at the time of CEUS. A follow-up ultrasound was also performed on three patients who underwent a DMSA scan, and a follow-up ultrasound confirmed the atrophy or cortical thinning remained in the area where cortical defects were observed in the DMSA scan.

Discussion

…

Another case also showed diffusely increased parenchymal echotexture, but no focal lesions were found on CEUS, and parenchymal damage due to VUR was confirmed in the follow-up ultrasound. The missed lesions on CEUS may be explained by the poor contrast between the true lesions and hypoperfused renal parenchyma.

…

Another reason might be due to the operators' insufficient experience detecting the perfusion abnormality. The examinator will be able to see diffuse pathological changes only when they are familiar with the condition of normal renal perfusion in CEUS. It is necessary to accumulate more experience with diffuse renal perfusion abnormalities in the future.

…

A follow-up ultrasound after CEUS (490.0 ± 168.5 days) was further investigated to overcome an insufficient number of DMSA scan results and to compensate for this study's limitation. We tried to improve the reliability of the negative results of CEUS by adding cases with normal findings on follow-up ultrasound. Additionally, a CEUS false-negative case was found in a patient with VUR, further adding an explanation of the false-negative risk during the CEUS examination.

…

In this study, the participants were infants at 9.0 months (2.0 months – 61.0 months). So, these data could be regarded as an additional value to the safety of ultrasound contrast agents.

**Reviewer #2: CUES ultrasound represents an important avenue of future development for pediatric patients. The lack of radiation and sedation provide clear benefits to pediatric patients. While the study demonstrates concordance with DMSA scan in most patients, the sample size (5) does not allow for a strong assessment of specificity or sensitivity. The study provides further safety data in 21 pediatric patients given the absence of adverse events. The study adds evidence to safety and feasibility in pediatric patients.**

🡪 Thank you for your thoughtful comments. We fully agree with your comments.

A total of 72 patients with suspected febrile urinary tract infection underwent a renal ultrasound during the study period, of which 21 patients participated in this study. Because this study was approved only as a clinical trial for ultrasound contrast agent, it was difficult to obtain written consent from the subjects' parents, and as a result, the number of participants was small. However, the patients who participated in this study did not show any side effects, such as headache, nausea, or skin erythema, even though they were infants at 9.0 months of age (2.0 months – 61.0 months). Therefore, these data could be regarded as an additional value to the safety of ultrasound contrast agents. As you mentioned, the study is insufficient to recommend CEUS alone for the diagnosis of APN due to the small number of subjects; however, we think it will be useful for researchers interested in the role and safety of ultrasound contrast agents in the diagnosis of APN in pediatric UTI patients.

A total of six patients had a follow-up ultrasound instead of a DMSA scan after CEUS (490.0 ± 168.5 days), of which only one showed abnormal findings in the grayscale ultrasound, such as parenchymal atrophy. In this patient, the initial grayscale ultrasound showed diffusely increased parenchymal echotexture and slight pelvic dilatation of both kidneys, but no focal lesions were found on CEUS. A follow-up voiding cystourethrography in this patient revealed vesicoureteral reflux, and the parenchymal damage was confirmed in the follow-up ultrasound. Therefore, this case could be regarded as a false-negative case of CEUS.

As noted in the manuscript, CEUS may give false-negative results if there are diffuse renal parenchymal changes, as in this patient, and it might be due to the operators' insufficient experience detecting the perfusion abnormality. The examinator will be able to see diffuse pathological changes only when they are familiar with the condition of normal renal perfusion in CEUS. It is necessary to accumulate more experience with diffuse renal perfusion abnormalities in the future.

The remaining five patients showed normal ultrasound findings, which indirectly confirmed that there was no APN at the time of CEUS. A follow-up ultrasound was also performed on three patients who underwent a DMSA scan, and a follow-up ultrasound confirmed the atrophy or cortical thinning remained in the area where cortical defects were observed in the DMSA scan.

As you mentioned, it is problematic that not all patients got a DMSA scan, which leads to the selected view. A follow-up ultrasound after CEUS (490.0 ± 168.5 days) was further investigated to overcome an insufficient number of DMSA scan results and to compensate for this study's limitation. We tried to improve the reliability of the negative results of CEUS by adding cases with normal findings on follow-up ultrasound. Additionally, a CEUS false-negative case was found in a patient with VUR, further adding an explanation of the false-negative risk during the CEUS examination.

**🡪 We revised and added the manuscript as follows:**

Materials and Methods

Participants: A total of 72 patients with suspected febrile urinary tract infection underwent a renal ultrasound during the study period, of which 21 patients participated in this study.

Data Analysis: The same analysis of follow-up grayscale images was performed if the follow-up ultrasound was performed instead of a DMSA scan.

Results

A total of six patients had a follow-up ultrasound instead of a DMSA scan after CEUS (490.0 ± 168.5 days), of which only one showed abnormal findings in the grayscale ultrasound, such as parenchymal atrophy. In this patient, the initial grayscale ultrasound showed diffusely increased parenchymal echotexture and slight pelvic dilatation of both kidneys, but no focal lesions were found on CEUS. A follow-up voiding cystourethrography in this patient revealed vesicoureteral reflux, and the parenchymal damage was confirmed in the follow-up ultrasound. Therefore, this case could be regarded as a false-negative case of CEUS. The remaining five patients showed normal ultrasound findings, which indirectly confirmed that there was no APN at the time of CEUS. A follow-up ultrasound was also performed on three patients who underwent a DMSA scan, and a follow-up ultrasound confirmed the atrophy or cortical thinning remained in the area where cortical defects were observed in the DMSA scan.

Discussion

…

Another case also showed diffusely increased parenchymal echotexture, but no focal lesions were found on CEUS, and parenchymal damage due to VUR was confirmed in the follow-up ultrasound. The missed lesions on CEUS may be explained by the poor contrast between the true lesions and hypoperfused renal parenchyma.

…

Another reason might be due to the operators' insufficient experience detecting the perfusion abnormality. The examinator will be able to see diffuse pathological changes only when they are familiar with the condition of normal renal perfusion in CEUS. It is necessary to accumulate more experience with diffuse renal perfusion abnormalities in the future.

…

A follow-up ultrasound after CEUS (490.0 ± 168.5 days) was further investigated to overcome an insufficient number of DMSA scan results and to compensate for this study's limitation. We tried to improve the reliability of the negative results of CEUS by adding cases with normal findings on follow-up ultrasound. Additionally, a CEUS false-negative case was found in a patient with VUR, further adding an explanation of the false-negative risk during the CEUS examination.

…

In this study, the participants were infants at 9.0 months (2.0 months – 61.0 months). So, these data could be regarded as an additional value to the safety of ultrasound contrast agents.

6. PLOS authors have the option to publish the peer review history of their article ([what does this mean?](https://journals.plos.org/plosone/s/editorial-and-peer-review-process#loc-peer-review-history)). If published, this will include your full peer review and any attached files.


If you choose “no”, your identity will remain anonymous but your review may still be made public.


Do you want your identity to be public for this peer review? For information about this choice, including consent withdrawal, please see our [Privacy Policy](https://www.plos.org/privacy-policy).

Reviewer #1: No

Reviewer #2: No

[NOTE: If reviewer comments were submitted as an attachment file, they will be attached to this email and accessible via the submission site. Please log into your account, locate the manuscript record, and check for the action link "View Attachments". If this link does not appear, there are no attachment files.]

While revising your submission, please upload your figure files to the Preflight Analysis and Conversion Engine (PACE) digital diagnostic tool, <https://pacev2.apexcovantage.com/>. PACE helps ensure that figures meet PLOS requirements. To use PACE, you must first register as a user. Registration is free. Then, login and navigate to the UPLOAD tab, where you will find detailed instructions on how to use the tool. If you encounter any issues or have any questions when using PACE, please email PLOS at [figures@plos.org](mailto:figures@plos.org). Please note that Supporting Information files do not need this step.
