# Supplementary material for: Contrast-enhanced ultrasound for the diagnosis of acute pyelonephritis in pediatric patients with urinary tract infection: A feasibility study
Source: PLoS One. 2023 Apr 5;18(4):e0284016. doi: 10.1371/journal.pone.0284016 (PMC10075446; doi:10.1371/journal.pone.0284016)
Supplement: Attachment — Submitted filename: Response to Reviewers.docx [file pone.0284016.s002.docx]

PONE-D-22-08175R1

Contrast-enhanced ultrasound for the diagnosis of acute pyelonephritis in pediatric patients with urinary tract infection

PLOS ONE

Dear Dr. Lee,

Thank you for submitting your manuscript to PLOS ONE. After careful consideration, we feel that it has merit but does not fully meet PLOS ONE’s publication criteria as it currently stands. Therefore, we invite you to submit a revised version of the manuscript that addresses the points raised during the review process.

Please submit your revised manuscript by Jan 02 2023 11:59PM. If you will need more time than this to complete your revisions, please reply to this message or contact the journal office at [plosone@plos.org](mailto:plosone@plos.org). When you're ready to submit your revision, log on to <https://www.editorialmanager.com/pone/> and select the 'Submissions Needing Revision' folder to locate your manuscript file.

Please include the following items when submitting your revised manuscript:

- A rebuttal letter that responds to each point raised by the academic editor and reviewer(s). You should upload this letter as a separate file labeled 'Response to Reviewers'.
- A marked-up copy of your manuscript that highlights changes made to the original version. You should upload this as a separate file labeled 'Revised Manuscript with Track Changes'.
- An unmarked version of your revised paper without tracked changes. You should upload this as a separate file labeled 'Manuscript'.

If you would like to make changes to your financial disclosure, please include your updated statement in your cover letter. Guidelines for resubmitting your figure files are available below the reviewer comments at the end of this letter.

If applicable, we recommend that you deposit your laboratory protocols in [protocols.io](http://protocols.io/) to enhance the reproducibility of your results. Protocols.io assigns your protocol its own identifier (DOI) so that it can be cited independently in the future. For instructions see: <https://journals.plos.org/plosone/s/submission-guidelines#loc-laboratory-protocols>. Additionally, PLOS ONE offers an option for publishing peer-reviewed Lab Protocol articles, which describe protocols hosted on [protocols.io](http://protocols.io/). Read more information on sharing protocols at <https://plos.org/protocols?utm_medium=editorial-email&utm_source=authorletters&utm_campaign=protocols>.

We look forward to receiving your revised manuscript.

Kind regards,

Shuai Ren
Academic Editor
PLOS ONE

Journal Requirements:

Please review your reference list to ensure that it is complete and correct. If you have cited papers that have been retracted, please include the rationale for doing so in the manuscript text, or remove these references and replace them with relevant current references. Any changes to the reference list should be mentioned in the rebuttal letter that accompanies your revised manuscript. If you need to cite a retracted article, indicate the article’s retracted status in the References list and also include a citation and full reference for the retraction notice.

Additional Editor Comments (if provided):

The authors should address the concerns raised by reviewer # 3 and update the references as per request.


[Note: HTML markup is below. Please do not edit.]

Reviewers' comments:

Reviewer's Responses to Questions

**Comments to the Author**

1. If the authors have adequately addressed your comments raised in a previous round of review and you feel that this manuscript is now acceptable for publication, you may indicate that here to bypass the “Comments to the Author” section, enter your conflict of interest statement in the “Confidential to Editor” section, and submit your "Accept" recommendation.

Reviewer #1: All comments have been addressed

Reviewer #3: All comments have been addressed

2. Is the manuscript technically sound, and do the data support the conclusions?

The manuscript must describe a technically sound piece of scientific research with data that supports the conclusions. Experiments must have been conducted rigorously, with appropriate controls, replication, and sample sizes. The conclusions must be drawn appropriately based on the data presented.

Reviewer #1: Partly

Reviewer #3: Partly

3. Has the statistical analysis been performed appropriately and rigorously?

Reviewer #1: N/A

Reviewer #3: Yes

4. Have the authors made all data underlying the findings in their manuscript fully available?

The [PLOS Data policy](http://www.plosone.org/static/policies.action#sharing) requires authors to make all data underlying the findings described in their manuscript fully available without restriction, with rare exception (please refer to the Data Availability Statement in the manuscript PDF file). The data should be provided as part of the manuscript or its supporting information, or deposited to a public repository. For example, in addition to summary statistics, the data points behind means, medians and variance measures should be available. If there are restrictions on publicly sharing data—e.g. participant privacy or use of data from a third party—those must be specified.

Reviewer #1: Yes

Reviewer #3: Yes

5. Is the manuscript presented in an intelligible fashion and written in standard English?

PLOS ONE does not copyedit accepted manuscripts, so the language in submitted articles must be clear, correct, and unambiguous. Any typographical or grammatical errors should be corrected at revision, so please note any specific errors here.

Reviewer #1: Yes

Reviewer #3: Yes

6. Review Comments to the Author

Please use the space provided to explain your answers to the questions above. You may also include additional comments for the author, including concerns about dual publication, research ethics, or publication ethics. (Please upload your review as an attachment if it exceeds 20,000 characters)

**Reviewer #1:** They answered the questions according to their available data. Id they want to solidify their manuscript some additional data might be needed.

Thank you for your thoughtful comments. We chose the DMSA scan as a reference standard for comparison with contrast-enhanced ultrasound findings to detect the presence of APN. However, only a small number of DMSA results were included, so we added results with follow-up ultrasound through the revised manuscript.

Also, the intrinsic limitation of the US to detect the presence of APNs, the poor generalizability of diagnosing APNs with contrast-enhanced US, and the methodological problem of DMSA scans with varying times, which might be resolving lesions spontaneously, were raised and criticized. However, as you mentioned, we did our best to make as much evidence as possible based on our data, but more was needed to solve the problems raised.

Despite the limitations in this study, not only the typical APN findings seen in CEUS but also false-negative cases were included, which might be misdiagnosed in future clinical settings or research. The findings, such as the most visible time of the lesion and the safety of ultrasound contrast agents in infant patients, were also included and discussed. The results or cases in this study with young infant patients may be reference data for large-scale studies that will conduct CEUS testing in the future.

Therefore, we hope it will serve as primary or feasible data for future research on ultrasound examination using ultrasound contrast agents. In summary, we added and toned down the end of the manuscript title as “a feasibility test” considering the study's limitations and the hope for future research: “Title: Contrast-enhanced ultrasound for the diagnosis of acute pyelonephritis in pediatric patients with urinary tract infection: a feasibility study”.

**Reviewer #3:** The authors sought to identify renal cortical changes associated with acute pyelonephritis (APN) related to vesicoureteral reflux. A study such as this was done previously which showed contrast enhanced US to be woefully inaccurate by comparison to other modalities, such as DMSA, CT and MRI. (Majd M, Nussbaum Blask AR, Markle BM, Shalaby-Rana E, Pohl HG, Park JS, Chandra R, Rais-Bahrami K, Pandya N, Patel KM, Rushton HG. Acute pyelonephritis: comparison of diagnosis with 99mTc-DMSA, SPECT, spiral CT, MR imaging, and power Doppler US in an experimental pig model. Radiology. 2001 Jan;218(1):101-8. doi: 10.1148/radiology.218.1.r01ja37101. PMID: 11152787.) This study was performed with rigor. In all test subjects, UTI was strictly defined and confirmed, the presence of VUR was confirmed, and APN was confirmed by histology.

Thank you for your thoughtful comments. As you mentioned, DMSA scan is the best way to evaluate acute pyelonephritis and renal scarring [14, 15]. In the case of acute inflammation, the DMSA scan shows diminished uptake due to cortical vasoconstriction, obstruction of peritubular capillaries by inflammatory cells, and ischemia-related renal tubular dysfunction [15]. Renal scars also show cortical defects on DMSA scans due to permanent damage to the renal parenchyma [15].

The study you mentioned (Majd M et al., Radiology. 2001;218(1):101-8) was conducted in 2001 under rigorous conditions. At that time, there were many cases where APN could not be detected with grayscale ultrasound and color Doppler ultrasound technology (NOT contrast-enhanced ultrasound using ultrasound-specific contrast agent). The limitations of conventional ultrasound tools were the starting point of this study, so they were mentioned in the introduction and discussion of the manuscript.

CEUS is a new imaging tool with an ultrasound-specific contrast agent, Sonovue®, which was initially approved for the diagnostic imaging of liver tumors in adults and children by the FDA in the United States in 2016. Despite its off-label use, it is now increasingly used in European and Asian countries.

It can detect perfusion abnormalities by observing contrast enhancement in the renal parenchyma after injecting a microbubble contrast agent. Therefore, it can show hypoperfusion areas more objectively than other ultrasound modalities (e.g., grayscale, color Doppler, power Doppler) and is less sensitive to patient movement. In addition, no radiation exposure or sedation is required, and the ultrasound contrast agent used for the examination is safe, making it very useful for pediatric patients.

We added the references, including as study you mentioned (Majd M et al. Radiology. 2001 Jan:218(1):101-8).

14. Majd M, Nussbaum Blask AR, Markle BM, Shalaby-Rana E, Pohl HG, Park JS, Chandra R, Rais-Bahrami K, Pandya N, Patel KM, Rushton HG. Acute pyelonephritis: comparison of diagnosis with 99mTc-DMSA, SPECT, spiral CT, MR imaging, and power Doppler US in an experimental pig model. Radiology. 2001;218(1):101-8.

15. Ataei N, Madani A, Habibi R, Khorasani M. Evaluation of acute pyelonephritis with DMSA scans in children presenting after the age of 5 years. Pediatr Nephrol. 2005;20(10):1439-1444.

With this high bar set, the proposed manuscript leaves some questions.

First, only 40% of infants with APN have positive DMSA renal scans, so the inclusion of only 42 patients may not have been sufficiently powered to tell the difference between the two studies. It is suggestive that the kappa for agreement between CEUS and DMSA scan is 0.8; however, are we certain that these results are generalizable?

Thank you for your thoughtful comments. Through this study, we tried to evaluate the diagnostic ability of CEUS compared to the DMSA scan, but as you mentioned, not all DMSA scans were done in time, and the number of participants was small. Since both acute pyelonephritis and renal scarring show diminished uptake on the DMSA scan, the SNMMI guideline updated in 2022 recommends performing the test within two weeks of an acute infection episode when performing a DMSA scan to diagnose acute pyelonephritis [19]. We agreed with your opinion, but as mentioned in the Limitation section in our manuscript, it was difficult to recommend an additional examination when no abnormal findings were found on imaging tests in patients with febrile UTI. This is one reason for the lack of prospective CEUS studies for the diagnosis of APN in pediatric patients.

We also agree that the generalization of the study results is difficult due to the small number of participants. Although the study was approved for using ultrasound-specific contrast agents, obtaining consent from parents was difficult, so sufficient participants could not be secured. However, we did our best to make as much evidence as possible based on our data, such as the addition of the result of follow-up ultrasound, but more was needed to solve the problems raised.

Despite the limitations in this study, not only the typical APN findings seen in CEUS but also false-negative cases were included, which might be misdiagnosed in future clinical settings or research. The findings, such as the most visible time of the lesion and the safety of ultrasound contrast agents in infant patients, were also included and discussed. The results or cases in this study with young infant patients may be reference data for large-scale studies that will conduct CEUS testing in the future.

🡪 Therefore, we hope it will serve as primary or feasible data for future research on ultrasound examination using ultrasound contrast agents. In summary, we added and toned down the end of the manuscript title as “a feasibility test” considering the study's limitations and the hope for future research: “Title: Contrast-enhanced ultrasound for the diagnosis of acute pyelonephritis in pediatric patients with urinary tract infection: a feasibility study”.

Also, we added the following reference:

19. Vali, R., Armstrong, I.S., Bar-Sever, Z. et al. SNMMI procedure standard/EANM practice guideline on pediatric [99mTc]Tc-DMSA renal cortical scintigraphy: an update. Clin Transl Imaging 2022;10:173–184.

Secondly, another significant methodological problem in this paper is that DMSA scans were done 137 +/- 166 days following the suspected UTI presentation which might have been sufficient time for some APN lesions to resolve spontaneously.

Thank you for your thoughtful comments. In the five patients who underwent DMSA scans, the mean time from ultrasound examination to DMSA scan was 137.2 ± 166.6 days (2, 3, 6, 287, and 388 days, respectively). In the patient who underwent a DMSA scan after 287 days, there were multiple cortical defects in the right kidney, and contrast-enhanced CT taken at the same time revealed multifocal cortical thinning, indirectly suggesting the presence of prior APN. In another case, the patient who underwent a DMSA scan after 388 days, there were no abnormal findings in the DMSA scan, and a follow-up grayscale ultrasound also revealed no renal scarring.

However, as you mentioned, renal scars do not appear in all APN patients [20], so APN may have resolved without sequelae through appropriate treatment in this patient. Therefore, the possibility of a false negative CEUS result cannot be ruled out. For these reasons, follow-up ultrasound after CEUS (490.0 ± 168.5 days) was further investigated to overcome an insufficient number of DMSA scan results and to improve the reliability of the negative results of CEUS. Follow-up ultrasound was performed in 6 patients with negative CEUS results, and there was no evidence of renal scarring on follow-up ultrasound except for one patient with bilateral grade 5 VUR. Although evaluation is limited because follow-up ultrasound was not performed in all patients and renal scarring does not always occur in patients with APN, this may suggest that CEUS has a high negative predictive value for diagnosing APN in febrile UTI patients.

We added the Limitation section of the Discussion as following paragraph:

“Second, not all DMSA scans were done in time [19]. In this study's five patients who underwent DMSA scans, the mean time from ultrasound examination to DMSA scan was 137.2 ± 166.6 days (2, 3, 6, 287, and 388 days, respectively). In the patient who underwent a DMSA scan after 388 days, there were no abnormal findings in the DMSA scan. However, renal scars do not appear in all APN patients [20], so APN may have resolved without sequelae through appropriate treatment in this patient. Therefore, the possibility of a false negative CEUS result cannot be ruled out.”

19. Vali, R., Armstrong, I.S., Bar-Sever, Z. et al. SNMMI procedure standard/EANM practice guideline on pediatric [99mTc]Tc-DMSA renal cortical scintigraphy: an update. Clin Transl Imaging. 2022;10:173–184.

20. Faust WC, Diaz M, Pohl HG. Incidence of post-pyelonephritic renal scarring: a meta-analysis of the dimercapto-succinic acid literature. J Urol. 2009;181(1):290-298.

I think the authors need to address these two concerns and updated the references to include prior work done.

We really appreciate your constructive comments and additional suggestions. Despite these limitations, this study is meaningful because it is the first prospective study to evaluate the feasibility of CEUS. Also, there were no adverse events in 21 patients of infant age who underwent CEUS in this study, which may add evidence for the safety of ultrasound contrast agents in pediatric patients. CEUS is a test with many advantages and is expected to play an essential role in diagnosing acute pyelonephritis in pediatric patients in the future.

Despite the limitations in this study, not only the typical APN findings seen in CEUS but also false-negative cases were included, which might be misdiagnosed in future clinical settings or research. The findings, such as the most visible time of the lesion and the safety of ultrasound contrast agents in infant patients, were also included and discussed. The results or cases in this study with young infant patients may be reference data for large-scale studies that will conduct CEUS testing in the future.

We hope this study will help explain the feasibility and safety of CEUS in diagnosing APNs in pediatric patients.

7. PLOS authors have the option to publish the peer review history of their article ([what does this mean?](https://journals.plos.org/plosone/s/editorial-and-peer-review-process#loc-peer-review-history)). If published, this will include your full peer review and any attached files.


If you choose “no”, your identity will remain anonymous but your review may still be made public.


**Do you want your identity to be public for this peer review?** For information about this choice, including consent withdrawal, please see our [Privacy Policy](https://www.plos.org/privacy-policy).

Reviewer #1: No

Reviewer #3: No

[NOTE: If reviewer comments were submitted as an attachment file, they will be attached to this email and accessible via the submission site. Please log into your account, locate the manuscript record, and check for the action link "View Attachments". If this link does not appear, there are no attachment files.]

While revising your submission, please upload your figure files to the Preflight Analysis and Conversion Engine (PACE) digital diagnostic tool, <https://pacev2.apexcovantage.com/>. PACE helps ensure that figures meet PLOS requirements. To use PACE, you must first register as a user. Registration is free. Then, login and navigate to the UPLOAD tab, where you will find detailed instructions on how to use the tool. If you encounter any issues or have any questions when using PACE, please email PLOS at [figures@plos.org](mailto:figures@plos.org). Please note that Supporting Information files do not need this step.
